# Minimum Detectable Change of Visual Acuity Measurements Using ETDRS Charts (Early Treatment Diabetic Retinopathy Study)

**DOI:** 10.3390/ijerph18157876

**Published:** 2021-07-25

**Authors:** María Carmen Sánchez-González, Raquel García-Oliver, José-María Sánchez-González, María-José Bautista-Llamas, José-Jesús Jiménez-Rejano, Concepción De-Hita-Cantalejo

**Affiliations:** 1 Department of Physics of Condensed Matter, Optics Area, University of Seville, 41012 Seville, Spain; raquelgobm@gmail.com (R.G.-O.); jsanchez80@us.es (J.-M.S.-G.); mbautista5@us.es (M.-J.B.-L.); mhita@us.es (C.D.-H.-C.); 2 Physiotherapy Department, Faculty of Nursing, Physiotherapy and Podiatry, University of Seville, 41009 Seville, Spain; jjjimenez@us.es

**Keywords:** ETDRS, minimal detectable change, visual acuity

## Abstract

In our work, we determined the value of visual acuity (VA) with ETDRS charts (Early Treatment Diabetic Retinopathy Study). The purpose of the study was to determine the measurement reliabilities, calculating the correlation coefficient interclass (ICC), the value of the error associated with the measure (SEM), and the minimal detectable change (MDC). Forty healthy subjects took part. The mean age was 23.5 ± 3.1 (19 to 26) years. Visual acuities were measured with ETDRS charts (96% ETDRS chart nº 2140) and (10% SLOAN Contrast Eye Test chart nº 2153). The measurements were made (at 4 m) under four conditions: Firstly, photopic conditions with high contrast (HC) and low contrast (LC) and after 15 min of visual rest, mesopic conditions with high and low contrast. Under photopic conditions and high contrast, the ICC = 0.866 and decreased to 0.580 when the luminosity and contrast decreased. The % MDC in the four conditions was always less than 10%. It was minor under photopic conditions and HC (5.83) and maximum in mesopic conditions and LC (9.70). Our results conclude a high reliability of the ETDRS test, which is higher in photopic and high contrast conditions and lower when the luminosity and contrast decreases.

## 1. Introduction

Visual acuity (VA) allows for the evaluation of the integrity of the central and peripheral visual pathway, detect refractive errors and control the progression of eye diseases [1]. There are many tests for VA measurement. Currently, for research purposes, test symbols based on the Logarithm of the Minimum Angle of Resolution (LogMAR) design are used, along with the Bailey–Lovie visual acuity chart [2], including the version produced by Ferris et al. [3] and the Early Treatment Diabetic Retinopathy Study (ETDRS) test [4].

The ETDRS visual acuity charts have five letters in each row and a uniform space between each letter and each row of letters. This is currently considered by many authors as the gold standard of visual acuity testing in research [4]. However, Solomon et al. [5] along with a narrative history of the evolution, modification, and legacy of the ETDRS classification system concluded that new updated must be implemented on the test.

One of the main objectives of a VA measurement is the detection of clinical changes in the subject. The reliability of a visual acuity measurement is important in the assessment of a subject, because it allows for the differentiation of whether a change in visual acuity represents a clinical change or corresponds to a measurement error. Any measurement method has associated systematic errors. Hence, small variations could not be attributed to changes in a patient’s health status.

The inherent error of the measurement, even when using standardized optotypes, can cause a failure where a true change in the VA, a false negative or a change in diagnosis when in fact there is none, or a false positive are not detected [6]. Computerized repetition and an average visual acuity measurement reduces test–retest variability [6,7]. A computer is used to produce random stimuli, along with automatic processing and statistical analysis of the subject’s response, which is not possible using printed graphics. In clinic, this is usually referred to as repeatability, usually calculated as the test–retest variability (TRV). For the evidence diagnostics to be valid and have good discriminatory capacity, they must have high repeatability. The ability to detect true visual change decreases as the TRV increases within the acuity data [7].

Some studies have reviewed and evaluated the repeatability of best corrected visual acuity in the ETDRS tests of healthy adults [8] with retinal pathology [9,10], as well as in children [1,11], Other authors have compared the ETDRS test with other tests [4,12,13,14,15] and evaluated the test–retest reliability of the electronic visual acuity [16] algorithm of the Diabetic Retinopathy Study of Early Treatment (E-ETDRS) [17,18,19]. They have concluded that ETDRS provides high repeatability coefficients. The precision or sensitivity of an instrument or scales is defined as the measurement’s ability to distinguish small differences. The more accurate an instrument is, the more likely it is to also capture small changes, while an insensitive instrument will require big changes to show any changes in its scores.

The accuracy of a scale is usually measured by the standard error of the measurement (SEM), which determines a threshold, above which the change is considered real (or the minimal detectable change). The minimal detectable change (MDC) represents the minimal change in a score that reflects an actual clinical change and not a change by mis measurement [20].

In our work, we determined the value of VA with ETDRS in high and low contrast conditions, since visual function can remain normal in high contrast and be affected in low contrast [21]. The purpose of the study was to determine the measurement reliabilities, calculating the correlation coefficient interclass (ICC), the value of the error associated with the measure (SEM) and the minimal detectable change (MDC).

## 2. Materials and Methods

### 2.1. Design

This test–retest reliability study of ETDRS chart was conducted in accordance with the 2013 Helsinki Declaration (Fortaleza, Brazil). The study protocol was approved by the Virgen Macarena—Virgen del Rocio Hospital Institutional Review Board (code number 1260-N-18). Informed consent was obtained from all participants. The study procedure was conducted in the facilities of the University.

### 2.2. Subjects

Forty healthy subjects (40 eyes, 24 females and 16 males) took part in the study. The mean age was 23.5 ± 3.1 (19 to 26) years. Participants were University of Seville students, teachers and administration staff. They were invited to participate in the study through a mailing system sent to the pharmacy faculty university community. The inclusion criteria were: (I) visual acuity (VA) between +0.10 and −0.30 LogMAR (20/25 to 20/10 Snellen Scale) measured with a Snellen Optotype; (II) a stable refraction for at least one year, meaning a change ≤0.50 diopters in the spherical and cylindrical refraction; (III) had not worn soft or rigid gas permeable contact lenses within the previous 24 h, and (IV) had not used near electronic devices within the last six hours. Furthermore, the exclusion criteria included: (V) eye diseases, such as glaucoma and cataracts; (VI) out of the norm values of accommodation amplitude, negative relative accommodation, positive relative accommodation and monocular and binocular accommodative facility testing; (VII) pregnant or lactating participants, and (VIII) participants with disorders of the eye muscles (IX) participants with strabismus or nystagmus, or any other disorder affecting ocular fixation. The exclusion criteria were checked with a comprehensive optometry exam that included: slit lamp examination, intraocular pressure measurement, binocular vision, and accommodation assessment.

### 2.3. Study Procedure

From a total sample of 55 participants, 15 were excluded from the study due to the aforementioned criteria. Investigations were done monocularly. The eye to be measured was randomly selected. The random arrangement was (1:1) generated by an independent researcher using Epidat^®^ 4.2 software (Department of Health, Government of Galicia, Spain). The participants carried their glass correction, if needed, and the study eye was examined when the contralateral eye was occluded. Visual acuity examinations were repeated two hours after the first examination. The measurements were made by a single expert and licensed optometrist. All of the measurements obtained were performed in the same way for all of the participants.

Visual acuity was measured with ETDRS charts (96% ETDRS chart nº 2140) and (10% SLOAN Contrast Eye Test chart nº 2153) displayed in the standard Lighthouse Low Vision Products light box (Lighthouse Low Vision Products, Long Island City, NY, USA).

The measurements were made (at 4 m) under four conditions: Firstly, photopic conditions with high contrast (HC) and low contrast (LC) and after 15 min of visual rest, mesopic conditions with high contrast (HC) and low contrast (LC).

The room was lit using a halogen lamp, and the lighting conditions were measured with a Lux LCD Illuminance Meter (Precision Vision^®^, Woodstock, IL, USA) to obtain a photopic luminance level of 85 cd/m^2^ or a mesopic luminance level of 0.75 cd/m^2^ [1,8].

The charts were front-illuminated by two 40-watt fluorescent tubes. For the photopic VA measurements, the room was lit. For the mesopic VA measurements, the lights in the room were off during the entire test.

The patient was asked to read the letter starting at the top row and continuing to the next row until they incorrectly identified a complete row, at which point the test was terminated [8].

The letter scores were converted to the LogMAR equivalent using the formula: LogMAR = 1.7 − (0.02) · (letter score) [17].

### 2.4. Statistical Analysis

Statistical analysis was carried out with SPSS statistics 25.0 (IBM Corporation, Armonk, NY, USA). Intraclass correlation coefficients (ICCs) were calculated on the basis of a two-way mixed model with an absolute agreement for a single rater/measurement [22]. Additionally, ICC_3,1_ were based on a two-way mixed model with a consistency agreement for single rater/measurement and ICC_1,1_ were based on one-way random effects model with an absolute agreement for a single rater/measurement being calculated to verify the first ICC. In all of the cases, the average measurements were obtained. The criteria for the values of the ICCs were as follows: >0.80, excellent reliability; 0.61–0.80, good reliability; 0.41–0.60, moderate reliability; and ≤0.40, poor reliability [23].

The standard error measurement (SEM) is an index that can be used to define the difference needed between the separate measures on a subject in order for the difference in the measures to be considered real. SEMs were calculated as arranged by Weir as [24]; $SEM=SD\sqrt{1-ICC}$, where SD was the pooled standard deviation from all of the measurements and ICC was a two-way mixed model with an absolute agreement for a single rater/measurement intraclass correlation coefficient. Minimal detectable change (MDC) can be defined as the minimal difference needed for a change to be considered real. MDC was calculated as follows: $MDC=SEM\times 1.96\times \sqrt{2}$. In addition, the MDC percentage was also calculated according to the following formula: $MDC_{\%}=\left(\frac{MDC}{mean}\right)\times 100$. An MDC % score of ≤30% was acceptable, whereas, an MDC% score of ≤10% was excellent. Bland–Altman plots were constructed with 95% limits of agreement (LOA) to represent the agreement between the two ETDRS visual acuity measurements, where the differences in the two measurements were compared to the visual acuity measurement mean. The LOA were assessed as the mean change ± 1.96 × SD of the difference [25].

## 3. Results

Eighty measurements from 40 eyes (40 participants) were included in the following results. The ETDRS visual acuity mean, standard deviation and range were expressed in LogMAR unit and letter scores. Furthermore, ICC, ICC_3,1_, ICC_1,1_, SEM, MDC, and MDC_%_ were also reported for all conditions.

### 3.1. Photopic and High Contrast

The first measurements obtained were 0.0065 ± 0.01607 (−0.22 to +0.20) LogMAR/84.67 ± 5.08 (75 to 96) letters and −0.0190 ± 0.01504 (−0.20 to +0.20) LogMAR/85.95 ± 4.75 (75 to 95) letters in the second measurements. ICC, ICC_3,1_, and ICC_1,1_ were 0.866, 0.893, and 0.864, respectively. All of the ICC values obtained excellent reliability results. SEM was 0.0361 LogMAR (1.80 letters score). MDC reported a change of 0.100 LogMAR (4.98 letters score) and the MDC percentage was 5.83%, obtaining an excellent score in MDC_%_. The Bland–Altman plot difference between the measurements was 0.0178, upper LOA was 0.08836 and lower LOA was −0.05276.

### 3.2. Photopic and Low Contrast

The first measurements obtained were 0.0130 ± 0.01563 (−0.20 to +0.22) LogMAR/84.35 ± 4.94 (74 to 95) letters and 0.0018 ± 0.01421 (−0.20 to +0.24) LogMAR/84.91 ± 4.49 (73 to 95) letters in the second measurements. ICC, ICC_3,1_, and ICC_1,1_ were 0.777, 0.779, and 0.777, respectively. All of the ICC values obtained good reliability results. SEM was 0.0444 LogMAR (2.21 letters score). MDC reported a change of 0.123 LogMAR (6.12 letters score) and MDC percentage was 7.23%, obtaining an excellent score in MDC_%_. The Bland–Altman plot difference between the measurements was 0.0179, upper LOA was 0.10512 and lower LOA was −0.06932.

### 3.3. Mesopic and High Contrast

The first measurements obtained were −0.0060 ± 0.01492 (−0.24 to +0.20) LogMAR/85.30 ± 4.71 (75 to 97) letters and −0.0060 ± 0.01427 (−0.20 to +0.24) LogMAR/85.30 ± 4.51 (73 to 95) letters in the second measurements. ICC, ICC_3,1_, and ICC_1,1_ were 0.733, 0.728, and 0.734, respectively. All of the ICC values obtained good reliability results. SEM was 0.0474 LogMAR (2.36 letters score). MDC reported a change of 0.131 LogMAR (6.54 letters score) and the MDC percentage was 7.66%, obtaining an excellent score in MDC_%_. The Bland–Altman plot difference between the measurements was 0.0075, upper LOA was 0.10158 and lower LOA was −0.08658.

### 3.4. Mesopic and Low Contrast

**Figure 1:**
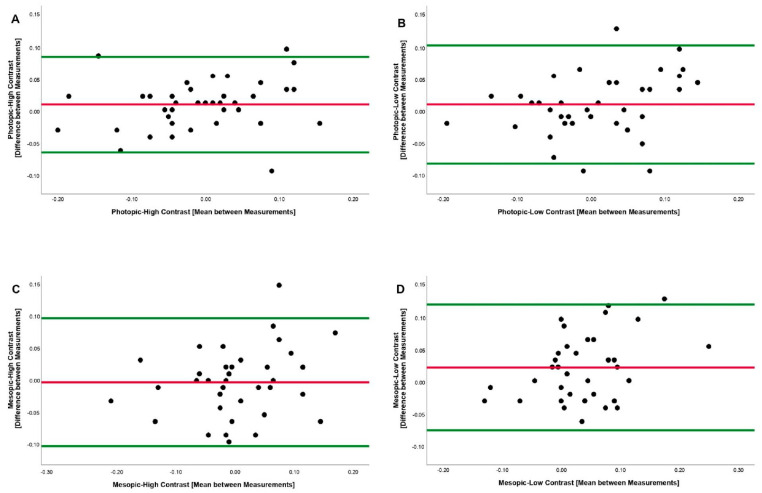
Bland–Altman plots between the measurements of VA. (**A**) Photopic and High Contrast; (**B**) Photopic and Low Contrast; (**C**) Mesopic and High Contrast; (**D**) Mesopic and Low Contrast. The central line indicates the mean between measurements. The external lines indicate limit of the 95 % agreement superior and inferior.

The first measurements obtained were 0.0565 ± 0.01492 (−0.18 to +0.28) LogMAR/82.17 ± 4.71 (71 to 94) letters and −0.0225 ± 0.01315 (−0.16 to +0.22) LogMAR/83.87 ± 4.15 (74 to 93) letters in the second measurements. ICC, ICC_3,1_, and ICC_1,1_ were 0.580, 0.617, and 0.567, respectively. All of the ICC values obtained moderate, good and moderate reliability results, respectively. SEM was 0.0583 LogMAR (2.91 letters score). MDC reported a change of 0.161 LogMAR (8.06 letters score) and the MDC percentage was 9.70% obtaining an excellent score in MDC_%_. The Bland–Altman plot difference between the measurements was 0.0292, upper LOA was 0.12151 and lower LOA was −0.06311. (Figure 1).

## 4. Discussion

The purpose of this study was to calculate the minimal detectable change (MDC) and examine the test–retest reliability of the Early Treatment Diabetic Retinopathy Study (ETDRS) in four different conditions of luminosity and contrast, along with the photopic and mesopic high (HC) and low contrast (LC). This study was conducted in healthy subjects with excellent VA due reproducibility in eyes with ocular disease or poor optical quality of the image would be affected.

In the review carried out, we found studies evaluating the test–retest reliability of (ETDRS) [1,6,8,9,10,11,13]. However, any measurement process has associated systematic errors in the measured variables, and therefore, changes smaller than the magnitude of the error cannot be attributed to changes in a patient’s health status.

In our work, we calculated the values of the error associated with the measurements (SEM) and the minimal detectable change (MDC), as they allowed us to determine whether an observed change between two measurements corresponded to a real change in the subject [20]. We also calculated the interclass correlation coefficients (ICC) to compare reliability between the measurements [24]. To our knowledge, ETDRS has only been measured for repeatability and test–retest reliability and none of the existing papers have addressed the study of the MDC and SEM.

According to ICC values, ETDRS showed excellent test–retest reliability in all of the four measurement conditions. The ICCs can theoretically vary between 0 and 1.0, where an ICC of 0 indicates no reliability and an ICC of 1.0 indicates perfect reliability [24]. Under photopic conditions and high contrast, the ICC = 0.866 and decreased to 0.580 when the luminosity and contrast decreased.

Our test–retest reliability results, measured with the interclass correlation coefficient, were in line with other studies. Beck et al. [17] measured test–retest reliability at 4 or 3 m in luminosity conditions (85 to 105 cd/m^2^) and HC (98%) in three groups of subjects with different visual acuity (VA) values. The ICC values obtained were ICC = 0.86 (VA > 20/40), ICC = 0.87 (VA 20/40 to 20/100), and ICC = 0.95 (VA < 20/100). In all of the cases, the value of the ICC was high, although the variability was slightly higher in participants with low visual acuity values. Camparini et al. [26] demonstrated values of 0.94 when measured in standard lighting conditions at 4 m.

Barrio et al. [8] determined the high repeatability of the test in far and near mesopic conditions (0.75 cd/m^2^), in both high (96%) and low contrast (10%). Similar repeatability was observed in HC and LC in both far (±0.11 logMAR) and near (±0.16 logMAR/±0.15 logMAR) conditions. Chaikitmongkol et al. [9] compared the repeatability of the ETDRS number charts, ETDRS Landolt C and ETDRS alphabet charts in four groups of subjects (healthy and with eye pathologies). They concluded that the ETDRS number chart was the one with the most repeatability in the measurement of VA for clinical practice. Patel et al. [10] described the repeatability between sessions of visual acuity measurements obtained with TDRS in patients with age-related macular degeneration (AMD). The measurements of patients with small druse were the most repeatable (coefficient of repeatability [CR] = 9 letters), and the measurements of patients with late AMD were the least repeatable (CR = 17 letters). This variability between the measurements may be due to the eye pathology itself.

The ICC refers to how repeated measurements vary in subjects, and a smaller variation indicates a higher reliability [27]. As in our work, in all the studies reviewed, high ICC values suggested a high reliability of the ETDRS test.

In addition, we calculated the value of the standard measurement error (SEM) and the minimal detectable change (MDC) as reliability indexes.

However, current literature lacks data on other errors in the measurement of VA with ETDRS with which to compare our results. Still, the % MDC in the four conditions was always less than 10%. It was minor under photopic conditions and HC (5.83) and maximum in mesopic conditions and LC (9.70). In addition, the number of letters that the subject failed increased as the luminosity and contrast decreased, as determined by SEM error values that progressively increased from 1.80 letters under photopic conditions and high contrast to 2.91 under mesopic conditions and low contrast.

The SEM and % MDC values were low, indicating that absolute reliability was high overall and the random measurement error was small. These values strengthen the high reliability of the ETDRS test, as referred to by other authors who measured only its repeatability [8,9,10,17,26]. Within the future research of lines, a new approach with an automated-ETDRS in near and intermediate distance [28] or a comparative assessment between Snellen scale and ETDRS [29] could improve scientific literature about ETDRS.

## 5. Conclusions

Our results concluded that a high reliability of the ETDRS test, which is higher in photopic and high contrast conditions and lower when the luminosity and contrast decreases.

With respect to clinical implications, a change in a VA score greater than the value of the MDC should be considered as a real change with 95% certainty. Therefore, we feel that the MDC value should be used in clinical research when evaluating VA to know exactly the score of each patient.

## Data Availability

The data presented in this study are available on request from the corresponding author.
